# [Pt(*O*,*O*′-acac)(γ-acac)(DMS)] Induces Autophagy in Caki-1 Renal Cancer Cells

**DOI:** 10.3390/biom9030092

**Published:** 2019-03-06

**Authors:** Giovanna Antonaci, Luca Giulio Cossa, Antonella Muscella, Carla Vetrugno, Sandra Angelica De Pascali, Francesco Paolo Fanizzi, Santo Marsigliante

**Affiliations:** 1 Laboratory of Cell Physiology, Department of Biological and Environmental Sciences and Technologies (Di.S.Te.B.A.), University of Salento, 73100 Lecce, Italy; giovanna.antonaci@unisalento.it (G.A.); lucagiulio.cossa@gmail.com (L.G.C.); santo.marsigliante@unisalento.it (S.M.); 2 Laboratory of Cell Pathology, Department of Biological and Environmental Sciences and Technologies (Di.S.Te.B.A.), University of Salento, 73100 Lecce, Italy; carlottavetrugno@virgilio.it; 3 Laboratory of General Inorganic Chemistry, Department of Biological and Environmental Sciences and Technologies (Di.S.Te.B.A.), University of Salento, 73100 Lecce, Italy; sandra.depascali@unisalento.it

**Keywords:** autophagy, cancerous renal cells, apoptosis, cisplatin

## Abstract

We have demonstrated the cytotoxic effects of [Pt(*O*,*O*′-acac)(γ-acac)(dimethyl sulfide (DMS))] on various immortalized cell lines, in primary cultures, and in murine xenograft models in vivo. Recently, we also showed that [Pt(*O*,*O*′-acac)(γ-acac)(DMS)] is able to kill Caki-1 renal cells both in vivo and in vitro. In the present paper, apoptotic and autophagic effects of [Pt(*O*,*O*′-acac)(γ-acac)(DMS)] and cisplatin were studied and compared using Caki-1 cancerous renal cells. The effects of cisplatin include activation of caspases, proteolysis of enzyme poly ADP ribose polymerase (PARP), control of apoptosis modulators B-cell lymphoma 2 (Bcl-2), Bcl-2-associated X protein (Bax), and BH3-interacting domain death agonist (Bid), and cell cycle arrest in G2/M phase. Conversely, [Pt(*O*,*O*′-acac)(γ-acac)(DMS)] did not induce caspase activation, nor chromatin condensation or DNA fragmentation. The effects of [Pt(*O*,*O*′-acac)(γ-acac)(DMS)] include microtubule-associated proteins 1A/1B light chain 3B (LC3)-I to LC3-II conversion, Beclin-1 and Atg-3, -4, and -5 increase, Bcl-2 decrease, and monodansylcadaverine accumulation in autophagic vacuoles. [Pt(*O*,*O*′-acac)(γ-acac)(DMS)] also modulated various kinases involved in intracellular transduction regulating cell fate. [Pt(*O*,*O*′-acac)(γ-acac)(DMS)] inhibited the phosphorylation of mammalian target of rapmycin (mTOR), p70S6K, and AKT, and increased the phosphorylation of c-Jun N-terminal kinase (JNK1/2), a kinase activity pattern consistent with autophagy induction. In conclusion, while in past reports the high cytotoxicity of [Pt(*O*,*O*′-acac)(γ-acac)(DMS)] was always attributed to its ability to trigger an apoptotic process, in this paper we show that Caki-1 cells die as a result of the induction of a strong autophagic process.

## 1. Introduction

Renal cell carcinoma (RCC) is among the top 10 most common cancers in the world; about 20%–30% of patients with RCC have metastasis to the first diagnosis, and over 95% of patients have multiple metastases [1]. Renal cell carcinoma resistance to conventional medical therapy remains the main obstacle to survival, and it is therefore important to develop new therapeutic strategies [2,3]. Indeed, many chemotherapeutic agents have been experienced in the treatment of RCC; among them, the cisplatin-based drugs are among the most common and effective chemotherapeutic agents for many types of human cancer [4]. However, patients that receive conventional platinum-based drugs also develop side effects and resistance to treatment, decreasing their effectiveness. 

The development of drugs targeting mammalian target of rapamycin (mTOR) (rapamycin analogs, i.e., temsirolimus and everolimus) has led to significant improvement in RCC prognosis. Although these new agents improve progression-free survival, none have shown a statistically significant improvement in overall survival. In effect, none are curative, and the duration of response is often limited [5]. In addition, based on clinical data, it has been argued that chronic drug exposure triggers the development of resistance, ultimately limiting the utility of mTOR inhibitor. Particularly, the mTOR-related proteins, AKT and S6K1, have been shown to be reactivated under long-term everolimus exposure [6].

In the search for new Pt complexes with better antitumor profiles that are less likely develop resistance, [Pt(*O*,*O*′-acac)(γ-acac)(dimethyl sulfide (DMS))] has been synthesized, a Pt(II) complex containing two acetyl groups (acac) and a sulfide ligand in the Pt coordination sphere [7,8]. Previous studies have shown that this new complex has a high and rapid cytotoxic activity, being able to induce apoptotic cell death in HeLa human endometrial carcinoma cells [9], in MCF-7 human breast cancer cells [10], in SH-SY5Y human neuroblastoma cells [11], and in ZL55 [12] and ZL34 [13] mesothelioma cells. Quite recently, we also showed that [Pt(*O*,*O*′-acac)(γ-acac)(DMS)] also has high and rapid citotoxicity on renal Caki-1 cells both in vitro and in vivo [14]. In these xenograft mice, [Pt(*O*,*O*′-acac)(γ-acac)(DMS)] also displayed potent antiangiogenic activity though the inhibition of vascular endothelial growth factor (VEGF) and matrix metalloproteinase-1 (MMP-1) expression in tumor tissues [14]. 

This is of interest since Caki-1 cells are intrinsically able to resist and inhibit apoptosis [15], whilst [Pt(*O*,*O*′-acac)(γ-acac)(DMS)] kills cells through a potent induction of apoptosis. Resistance to apoptosis may be due to various factors. During nephrogenesis, the transcription factor paired box protein 2 (PAX2) is differentially expressed depending upon the kidney development phase, and it is barely quantifiable in mature kidney. In Caki-1 cells, PAX2 is overexpressed and contributes to cisplatin resistance [16]. Furthermore, Caki-1 cells resist apoptosis through the secretion of IL-6 that suppresses the induction of apoptosis acting in an autocrine way [17]. Although [Pt(*O*,*O*′-acac)(γ-acac)(DMS)] most often activates the mitochondrial apoptotic pathway, another recent in vitro investigation of neuroblastoma cells has revealed that [Pt(*O*,*O*′-acac)(γ-acac)(DMS)] leads to both apoptosis and activation of autophagy [18].

Thus, the aim of the present paper is to investigate apoptotic and/or autophagic pathways caused by cisplatin and [Pt(*O*,*O*′-acac)(γ-acac)(DMS)] treatment in Caki-1 cells.

## 2. Materials and Methods

### 2.1. Cell Culture 

Caki-1 cells (human renal carcinoma cell line) were maintained in McCoy’s medium supplemented with 10% fetal bovine serum, penicillin/streptomycin, and 1% l-glutamine. All cell cultures were routinely grown in 75 cm^2^ cell culture flasks and were sustained at 37 °C, 5% CO_2_, and 95% relative humidity. Cells were grown to 70%–80% confluence and then treated with cisplatin and [Pt(*O*,*O*′-acac)(γ-acac)(DMS)] at various concentrations and for different incubation periods. 

### 2.2. Cytotoxicity Assay

To assess the cytotoxicity of [Pt(*O*,*O*′-acac)(γ-acac)(DMS)] and cisplatin, we used both 3-(4,5-dimethylthiazol-2-yl)-2,5-diphenol tetrazolium bromide (MTT) [19] and sulforhodamine B (SRB) [20] methods, as previously described. Viable cells were also counted using the trypan blue exclusion assay and light microscopy. The percentage of live cells was evaluated as the absorbance ratio of treated to untreated cells. The data presented are means ± standard deviation (SD) from eight replicate wells per microtiter plate.

In addition, cell death was then measured by quantifying the percentage of cells that exhibited annexin V-fluorescein isothiocyanate (FITC) and/or propidium iodide (PI) fluorescence using a flow cytometer. Total cell death was quantified by adding the percentage of cells detected in the upper left (PI), upper right (PI + annexin V-FITC), and lower right (annexin V-FITC) quadrants in the FACS dotplots (Becton-Dickinson, CA, USA).

### 2.3. Apoptosis Analysis

For the 4,6-diammine-2-phenylindol (DAPI) staining, cells treated with [Pt(*O*,*O*′-acac)(γ-acac)(DMS)] or cisplatin were fixed with 4% paraformaldehyde and incubated with 1 mg mL^−1^ DAPI in phosphate buffeed saline (PBS) for 15–20 min. Cells were mounted on glass slides and analyzed using fluorescence microscopy. For statistical analysis of each experiment, 5–10 fields (magnification 20×) were analyzed using the image analysis software ImageJ so as to detect only the nuclei with chromatin condensation. The mean ± SD was calculated and displayed as a bar graph. For the DNA fragmentation assay, genomic DNA from Caki-1 cells was prepared using the Wizard Genomic DNA Purification Kit (Promega, Milan, Italy) according to the manufacturer’s protocol. DNA was dissolved in Tris-ethylenediaminetetraacetic acid (EDTA) buffer, and 40 µg of DNA was separated on a 1.2% agarose gel containing 0.1 mg mL^−1^ ethidium bromide, visualized under ultraviolet light, and photographed.

### 2.4. Preparation of Subcellular Fraction

Preparation of subcellular fractions performed as previously reported [21].

### 2.5. Western Blotting Analysis

Western blotting analysis, immunodetection, and densitometric analysis of subcellular fraction were performed as previously described [21]. Briefly, lysates or subcellular samples were subjected to 8%–12% sodium dodecyl sulfate polyacrylamide gel electrophoresis (SDS-PAGE). The proteins were transferred to nitrocellulose by semi-dry transfer at 25 V for 1 h using a Trans-Blot SD apparatus (BioRad, Hercules, CA, USA). Membranes were blocked with PBS containing 5% milk and 0.1% Tween-20 at room temperature, and incubated with primary antibodies overnight at 4 °C. Membranes were then washed and incubated with horseradish peroxidase (HRP)-conjugated goat anti-rabbit or anti-mouse antibody (1:10,000) for 1 h. The signals were visualized with Enhanced Chemiluminescence detection solution (Amersham Pharmacia Biotech, Milan, Italy). The purity of fractions was tested by immunoblotting with β-actin. Densitometric analysis was carried out on the Western blots using the National Institute of Health (NIH) Image v1.63 software (National Institutes of Health, Bethesda, MD, USA). The pixel intensity for each region was analyzed, the background was subtracted, and the protein expressions were normalized to β-actin loading control for each lane.

### 2.6. Cell Cycle Analysis

Cell cycle analysis was performed using a FACSCanto flow cytometer (Becton-Dickinson, San Jose, CA, USA). After treatments, cells were washed with cold PBS and harvested by centrifugation. Then, cells were resuspended in cold absolute ethanol and stored at −20 °C overnight. RNase A (0.2 mg mL^−1^) and propidium iodide (20 µg mL^−1^) were added, followed by incubation for 40 min in the dark, and cell cycle distribution was analyzed by flow cytometry cell sorting. Cell cycle distribution (sub-G1, G0/G1, S, and G2/M phase fraction) was analyzed using FlowJo software (Ver. 7.6.5, TreeStar, Ashland, OR, USA).

### 2.7. Design and Preparation of Small Interfering RNAs

Small interfering RNAs (siRNAs) were prepared by an in vitro transcription method. Silencing of apoptosis inducing factor (AIF) by siRNA was performed as previously reported using sense (5′-CUUGUUCCAGCGAU GGCAUTT-3′) and antisense (5′-AUGCCAUCGCUGGAACAAGTT-3′) RNA oligos that target nucleotides 151–171 in human AIF [21]. All template oligonucleotides were chemically synthesized and purified by polyacrylamide gel electrophoresis. In vitro transcription, annealing, and purification of siRNA duplexes were performed using the protocol supplied with the T7 RiboMAX Express RNAi System (Promega, Milan, Italy), as previously described [21].

### 2.8. Small Interfering RNA Transfection

Caki-1 cells (50%–70% confluence) were transfected with siRNA duplexes using the protocol supplied with the siRNA transfection reagent sc-29528 (Santa Cruz Biotechnology, Santa Cruz, CA, USA). Briefly, 5 μL of siRNA duplex (i.e., 0.6 μg siRNA) was diluted into 100 μL siRNA transfection medium: sc-36868 (Santa Cruz Biotechnology) without serum and antibiotics for about 20 min to form a lipid–siRNA complex. Transfection was initiated by adding the lipid–siRNA complex to six-well plates. Cells were incubated for 6 h at 37 °C in a CO_2_ incubator. The final concentration of siRNA was 30 nM. Then, 1 mL of normal growth medium containing two times the normal serum and antibiotics concentration (2× normal growth medium) was added to the cells, without removing the transfection mixture, and incubated for an additional 24 h. Finally, the medium was aspirated and replaced with fresh 1× normal growth medium.

### 2.9. Visualization of Monodansylcadaverine 

Autophagic vacuoles were labeled with monodansylcadaverine (MDC) by incubating Caki-1 cells grown on coverslips with 50 µM MDC in PBS at 37 °C for 30 min. Cells were fixed in 4% paraformaldehyde and washed with PBS. The cellular fluorescent changes were observed through confocal microscopy Zeiss LSM 700. For densitometric analysis, the NIH Image (v1.63) software (National Institutes of Health) was used.

### 2.10. Statistical Analysis

Data, presented as means ± SD, were analyzed using GraphPad Prism 5 software (GraphPad Software, La Jolla, CA, USA) with unpaired Student’s *t*-test or one-way analysis of variance (ANOVA), and when this returned *p* < 0.05, a post hoc analysis using Bonferroni test was performed; we used the Bonferroni–Dunn post hoc test in the ANOVA after a significant omnibus *F*-test. *p* < 0.05 was accepted as the level of statistical significance. 

## 3. Results

### 3.1. Cytotoxicity and Flow Cytometric Analyses of [Pt(O,O′-acac)(γ-acac)(DMS)] and Cisplatin in Caki-1 Cells

The cytotoxicity data shown here were obtained by MTT metabolic assay and confirmed by SRB assay to rule out potential effects of [Pt(*O*,*O*′-acac)(γ-acac)(DMS)] on mitochondrial enzymes. Indeed, comparable results were obtained when cell number was directly determined by cell counting. Consequently, we used MTT assay in the reported combined experiments.

**Figure 1:**
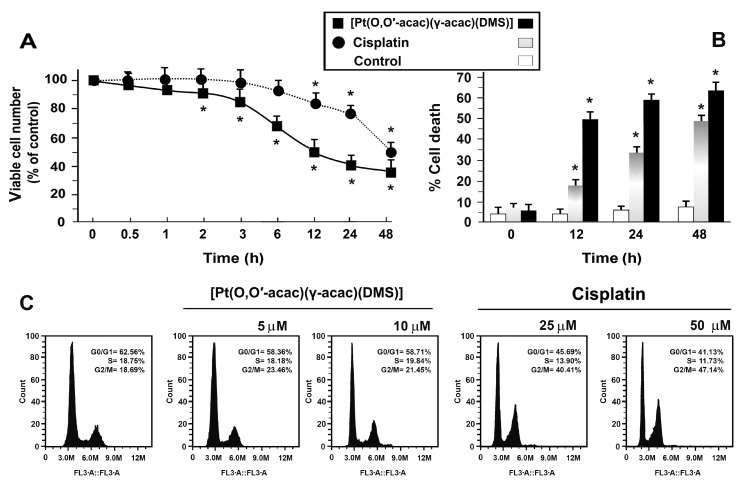
Cytotoxic effects of [Pt(*O*,*O*′-acac)(γ-acac)(DMS) and cisplatin and effects on the cell cycle of Caki-1 cells. Caki-1 cells were treated with 10 µM [Pt(*O*,*O*′-acac)(γ-acac)(dimethyl sulfide (DMS))] or with 50 µM cisplatin. Cell viability was monitored by 3-(4,5-dimethylthiazol-2-yl)-2,5-diphenol tetrazolium bromide (MTT) assay (**A**) and cell death was quantified by fluorescence-activated cell sorter (FACS) after propidium iodide (PI)/annexin V-fluorescein isothiocyanate (FITC) staining (**B**), over a period of 48 h. Data are means ± standard deviation (SD) of five independent experiments with eight replicates in each, and are presented as percent of control. * *p* < 0.01 between treated and untreated cells (white bar), by Student’s *t*-test. (**C**) Cells were treated with [Pt(*O*,*O*′-acac)(γ-acac)(DMS)] or cisplatin for 24 h, and cell cycle distribution was analyzed by flow cytometry after staining the cells with PI. Representative FACS histogram from six separate experiments is shown.

As previously shown, the cytotoxicity of [Pt(*O*,*O*′-acac)(γ-acac)(DMS)] is approximately 5-fold greater than that observed for cisplatin (for [Pt(*O*,*O*′-acac)(γ-acac)(DMS)], half maximal inhibitory concentration (IC_50_) 6.92 ± 0.084 µM; for cisplatin, IC_50_ 37.35 ± 0.5 µM). Here, a time course cytotoxicity curve was performed using 10 µM [Pt(*O*,*O*′-acac)(γ-acac)(DMS)] and 50 µM cisplatin; it was found that [Pt(*O*,*O*′-acac)(γ-acac)(DMS)] is more powerful than cisplatin (Figure 1A,B).

Interestingly, about 50% of cells died after only 12 h of treatment with 10 μM [Pt(*O*,*O*′-acac)(γ-acac)(DMS)], while it was necessary to incubate for about 48 h to get the same effect using 50 μM cisplatin (Figure 1A,B). Cell death, after cisplatin or [Pt(*O*,*O*′-acac)(γ-acac)(DMS)] treatment, was also measured by quantifying the percentage of cells that exhibited annexin V-FITC and/or PI fluorescence. It can be seen that cell death induced by [Pt(*O*,*O*′-acac)(γ-acac)(DMS)] was significantly higher than cell death due to cisplatin (Figure 1B). 

Flow cytometric analysis showed a cell cycle arrest in G2/M phase after treatment with 25 and 50 µM cisplatin, for 24 h; conversely [Pt(*O*,*O*′-acac)(γ-acac)(DMS)] (5 and 10 µM) did not induce variation in cell cycle profiling (Figure 1B).

### 3.2. [Pt(O,O′-acac)(γ-acac)(DMS)] Does Not Induce Apoptosis in Caki-1 Cells

**Figure 2:**
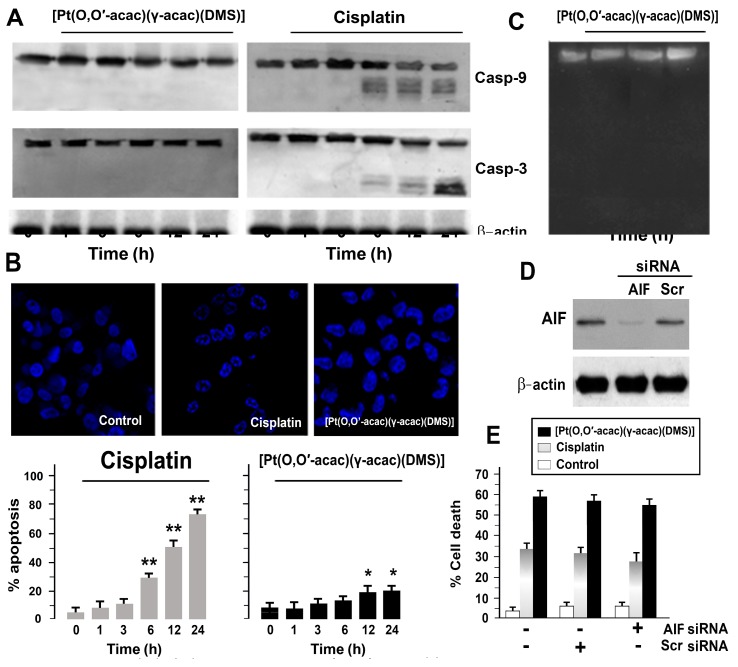
[Pt(*O*,*O*′-acac)(γ-acac)(DMS)] does not induce apoptosis in Caki-1 cells. (**A**) Expression of cleaved caspase-9 and -3 in Caki-1 cells. Cell were treated with 10 µM of [Pt(*O*,*O*′-acac)(γ-acac)(DMS)] or 50 µM of cisplatin for the indicated time, and then subjected to Western blotting. Incubation with anti-β-actin confirmed the equal protein loading. The results shown are representative of three different experiments. (**B**) (Up) Caki-1 cells were treated, or not, with cisplatin, or with [Pt(*O*,*O*′-acac)(γ-acac)(DMS)] for 24 h, and then stained with 4,6-diammine-2-phenylindol (DAPI); the representative fields by confocal microscopy (magnification 40×) of one of four independent experiments are shown. (Down) Quantification of the percentage of apoptotic nuclei obtained from cells stained with DAPI (means ± SD; *n* = 4), after treatment for different times with 10 µM [Pt(*O*,*O*′-acac)(γ-acac)(DMS)] or 50 µM cisplatin. ** *p* < 0.01 between cisplatin-treated and untreated cells and * *p* < 0.05 between [Pt(*O*,*O*′-acac)(γ-acac)(DMS)]-treated and untreated cells, by Student’s *t*-test. (**C**) Visualization of DNA fragmentation in [Pt(*O*,*O*′-acac)(γ-acac)(DMS)]-treated Caki-1 cells. Total DNA was isolated and separated on a 1% agarose gel. A representative example of three independent experiments is shown. (**D**,**E**) Caki-1 cells were transfected with 30 nM small interfering RNA (siRNA) oligos for apoptosis inducing factor (AIF) and then treated with 10 µM [Pt(*O*,*O*′-acac)(γ-acac)(DMS)]. (**D**) Immunoblotting detection of AIF in cell extracts 48 h after siRNA transfection using polyclonal anti-AIF antibody. Controls were provided by untransfected cells and cells transfected with scrambled siRNA oligos (Scr). Incubation with anti-β-actin confirmed the equal protein loading. A representative example of three independent experiments is shown. (**E**) Cell death was quantified by FACS after propidium iodide (PI)/annexin V-FITC staining, in transfected Caki-1 cells treated with 10 µM of [Pt(*O*,*O*′-acac)(γ-acac)(DMS)] or 50 µM cispltin for 24 h. Data are means ± SD of five independent experiments.

No caspase-9 and -3 proteolytic activation was observed in cells treated with 10 µM [Pt(*O*,*O*′-acac)(γ-acac)(DMS)], while 50 µM cisplatin induced caspase activation after 6 h (Figure 2A). Similarly, 10 µM [Pt(*O*,*O*′-acac)(γ-acac)(DMS)] did not induce chromatin condensation or DNA fragmentation (Figure 2B,C), whilst 50 µM cisplatin caused the appearance of the typical apoptotic nuclei in DAPI-stained cells (Figure 2B). Finally, no DNA fragmentation in [Pt(*O*,*O*′-acac)(γ-acac)(DMS)]-treated cells was observed (10 µM). Thus, these results suggest that in Caki-1 cells, [Pt(*O*,*O*′-acac)(γ-acac)(DMS)] cytotoxicity may not be due to apoptosis. 

It is known that nuclear translocation of AIF is induced by several death stimuli [17]; therefore, we evaluate the involvement of AIF using small interfering RNAs (siRNAs) for AIF, in [Pt(*O*,*O*′-acac)(γ-acac)(DMS)]-treated Caki-1 cells. As shown by immunoblotting detection of AIF in Caki-1 extracts, following treatment with AIF siRNA, AIF protein levels were significantly decreased (Figure 2D). Controls were provided by untransfected cells (Con) and cells transfected with scrambled siRNA oligos (Scr) (Figure 2D). The silencing of AIF did not lead to a significant decrease in cell death after cisplatin (*p* = 0.01) or [Pt(*O*,*O*′-acac)(γ-acac)(DMS)] (*p* = 0.06) treatment in Caki-1 cells (Figure 2E).

### 3.3. [Pt(O,O′-acac)(γ-acac)(DMS)] Induces Autophagy in Caki-1 Cells

**Figure 3:**
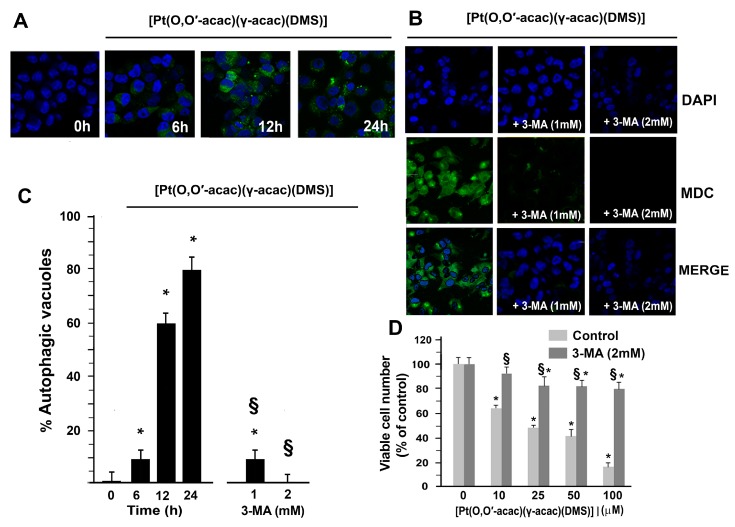
[Pt(*O*,*O*′-acac)(γ-acac)(DMS)] induces autophagy in Caki-1 cells. (**A**) Caki-1 cells were treated with 10 µM [Pt(*O*,*O*′-acac)(γ-acac)(DMS)] for the indicated times or (**B**) Caki-1 cells were pretreated, or not, with 1 mM of 3-MA or 2 mM of 3-MA, then treated with 10 µM [Pt(*O*,*O*′-acac)(γ-acac)(DMS)] for 24 h; subsequently, Caki-1 cells were stained with monodansylcadaverine (MDC) and 4′,6-diamidino-2-phenylindole (DAPI). The cellular fluorescent changes were observed through confocal microscopy Zeiss LSM 700 (Carl Zeiss AG, Oberkochen, Germany). (**C**) Quantification of the percentage of autophagic vacuoles are presented as means ± SD. * *p* < 0.001 between treated and untreated cells; ^§^ *p* < 0.001 between cells treated with 3-MA and [Pt(*O*,*O*′-acac)(γ-acac)(DMS)], and cells treated with [Pt(*O*,*O*′-acac)(γ-acac)(DMS)] alone, by Student’s *t*-test (*n* = 5). (**D**) Caki-1 cells were treated with increasing concentrations of [Pt(*O*,*O*′-acac)(γ-acac)(DMS)] and 2 mM of 3-MA for 24 h, and cell viability was monitored by MTT assay. * *p* < 0.001 between treated and untreated cells, by Student’s *t*-test (*n* = 5). ^§^ *p* < 0.001 between cells treated with SP600125 and [Pt(*O*,*O*′-acac)(γ-acac)(DMS)], and cells treated with [Pt(*O*,*O*′-acac)(γ-acac)(DMS)] alone; ^§^ *p* < 0.001 between cells treated with 3-MA and [Pt(*O*,*O*′-acac)(γ-acac)(DMS)], and cells treated with [Pt(*O*,*O*′-acac)(γ-acac)(DMS)] alone, by Student’s *t*-test (*n* = 5).

The lack of [Pt(*O*,*O*′-acac)(γ-acac)(DMS)]-induced apoptosis prompted us to study another mechanism of cell death, autophagy. Monodansylcadaverine may be used as a specific marker for autophagic vacuoles, since it accumulates in acidic compartments enriched in lipids, and staining intensity increases in cells induced to undergo autophagy. Caki-1 cells were then treated with 10 µM [Pt(*O*,*O*′-acac)(γ-acac)(DMS)] at different incubation times (6, 12, 24 h) and subsequently stained with MDC and DAPI (for nuclei localization). A time-dependent increase of fluorescence signals was evident in autophagic vacuoles of [Pt(*O*,*O*′-acac)(γ-acac)(DMS)]-treated cells (Figure 3A). Fluorescence intensities of autophagic vacuoles were drastically decreased by preincubating cells with crescent concentration of 3-methyladenine (3-MA), a specific inhibitor of autophagy (Figure 3B,C). Furthermore, the pretreatment with the autophagy inhibitor, 3-MA, or autophagolysosome fusion inhibitor significantly decreased cell death due to [Pt(*O*,*O*′-acac)(γ-acac)(DMS)] (Figure 3D). 

**Figure 4:**
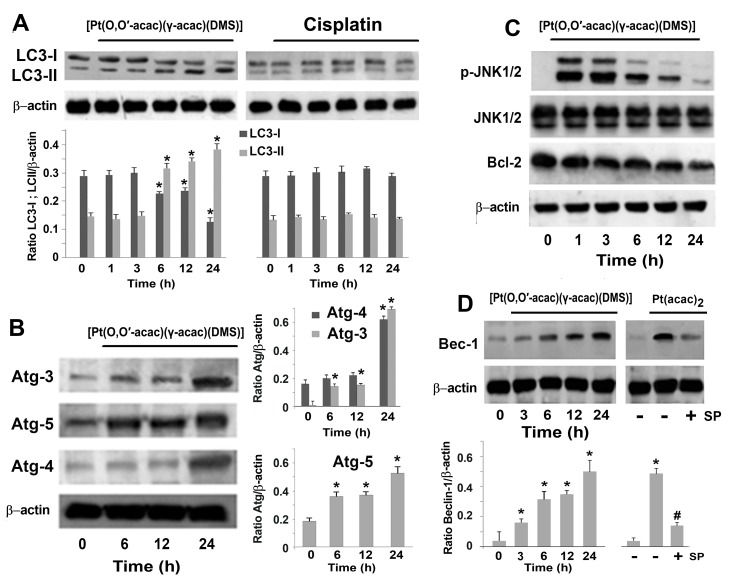
[Pt(*O*,*O*′-acac)(γ-acac)(DMS)] induces autophagy in Caki-1 cells. (**A**–**C**) Caki-1 cells were treated with 10 µM [Pt(*O*,*O*′-acac)(γ-acac)(DMS)] or with 50 µM cisplatin for different times. Cell lysates were analyzed by Western blotting using microtubule-associated proteins 1A/1B light chain 3B (LC3)I-II, autophagy-related (Atg)-3, Atg-4, Atg-5, JNK, and B-cell lymphoma 2 (Bcl-2) antibodies. Sequential incubation with anti-β-actin confirmed the equal protein loading. Representative immunoblots of three experiments are depicted. (**A**, down) Densitometric analysis of LC3I-II normalized to β-actin. (**B**, Right) Densitometric analysis of Atg-3, Atg-4, and Atg-5, normalized to β-actin. The data are means ± SD of three different experiments. * *p* < 0.001 between treated and untreated cells, by Student’s *t*-test (*n* = 3). (**D**) (Up) Cells, were incubated with 10 µM [Pt(*O*,*O*′-acac)(γ-acac)(DMS)] for the indicated time or were pretreated with 10 µM of JNK inhibitor SP600125 (SB) and then incubated with 10 µM [Pt(*O*,*O*′-acac)(γ-acac)(DMS)] (Pt(acaca)_2_)_,_ for 24 h. Cell lysates were analyzed by western blotting using monoclonal antibody specific to Beclin-1. Sequential incubation with anti-β-actin confirmed the equal protein loading. These figures are representative of five independent experiments. (Down) Densitometric analysis of Beclin-1 normalized to β-actin.

Thus, we analyzed the conversion of LC3-I to LC3-II, the active form of LC3-I, essential autophagic markers in the process of elongation and maturation of phagophore. Figure 4A shows that 10 µM [Pt(*O*,*O*′-acac)(γ-acac)(DMS)] induced LC3-I (19 kDa) to LC3-II (17 kDa) conversion in a time-dependent manner; no effects were seen when using 50 µM cisplatin (Figure 4A). During the nucleation phase of phagophore, Beclin-1, after detachment from Bcl-2, forms a complex with Vps34 facilitating the recruitment of Atg proteins. It is known that c-Jun N-terminal kinase (JNK)1/2 activation leads to Bcl-2 phosphorylation and Beclin-1 detachment [22]. Indeed, in cells treated with 10 µM [Pt(*O*,*O*′-acac)(γ-acac)(DMS)], we observed that Beclin-1 and Atg-3, -4, and -5 increased, Bcl-2 decreased, and JNK1/2 was activated by phosphorylation (Figure 4B–D). Preincubation of cells for 24 h with 10 µM SP600125, a JNK inhibitor, blocked the [Pt(*O*,*O*′-acac)(γ-acac)(DMS)]-induced Beclin-1 increase (Figure 4D). 

**Figure 5:**
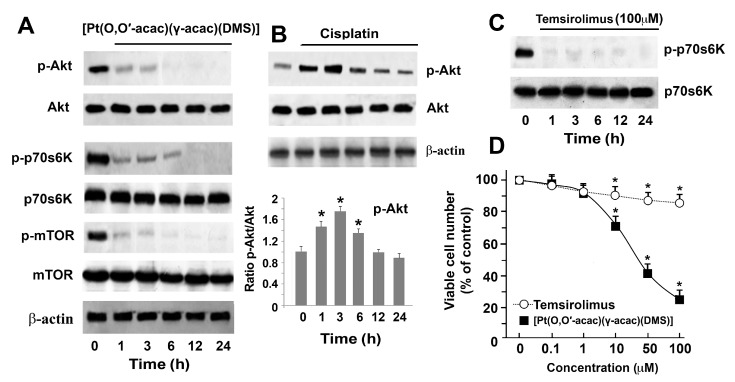
[Pt(*O*,*O*′-acac)(γ-acac)(DMS)] induces autophagy through inhibition of the phosphatidine 3-kinase (PI3K)/AKT-mTOR pathway. (**A**) Cell lysates obtained from Caki-1 cells treated with 10 µM [Pt(*O*,*O*′-acac)(γ-acac)(DMS)], for the indicated times, were subjected to Western blotting analyses with monoclonal antibodies specific to p-p70S6K, p70S6K, p-mTOR, m-TOR, p-AKT, and AKT. (**B**) (Up) Cell lysates from cells treated with 50 µM cisplatin, for the indicated times, were subjected to Western blotting analyses with monoclonal antibodies specific to p-AKT and AKT. Sequential incubation with anti-β-actin confirmed the equal protein loading. These figures are representative of six independent experiments. (Down) Densitometric analysis of p-AKT (normalized respectively to total AKT). (**C**) Caki-1 cells were treated, or not, with 100 µM temsirolimus for the indicated times and cell lysates were analyzed by Western blotting using antibodies specific to p-p70S6K and total p70S6K. The figure is representative of three independent experiments. (**D**) Caki-1 cells were treated, or not, with different concentrations of [Pt(*O*,*O*′-acac)(γ-acac)(DMS)] or temsirolimus for 24 h, and cell viability was monitored by MTT assay. * *p* < 0.001 between treated and untreated cells by Student’s *t*-test (*n* = 3).

We therefore analyzed the phosphatidine 3-kinase (PI3K)/AKT-mTOR-p70S6K pathway in Caki-1 cells treated with [Pt(*O*,*O*′-acac)(γ-acac)(DMS)]. Figure 5A shows that 10 µM [Pt(*O*,*O*′-acac)(γ-acac)(DMS)] inhibited the phosphorylation of mTOR already after 1 h treatment, thus promoting the inhibition of p70S6K phosphorylation. Consistently with previous data showing a functional relationship between mTOR and PI3K/AKT [23], we also showed that 10 µM [Pt(*O*,*O*′-acac)(γ-acac)(DMS)] inhibited, in a time-dependent manner, the phosphorylation of AKT (Figure 5A). Conversely, 50 µM cisplatin had no effect on phosphorylation of AKT (Figure 5B). We also compared the cytotoxic effects of [Pt(*O*,*O*′-acac)(γ-acac)(DMS)] to those exerted by temsirolimus, a soluble ester of rapamycin and a natural product and inhibitor of mammalian target of rapamycin (mTOR) [24]. Thus, Caki-1 cells were treated with 100 µM temsirolimus for various times (1, 3, 6, 12, and 24 h) and then cell lysates were analyzed by Western blotting with antibodies against p-p70S6K and total p70S6K. It can be seen that temsirolimus abolished the phosphorylation of p70S6K at all experimental points (Figure 5C). Furthermore, Caki-1 cell viability was assessed in cells treated, or not, with different concentrations of [Pt(*O*,*O*′-acac)(γ-acac)(DMS)] or temsirolimus. We show that temsirolimus had a significant cytotoxicity starting at 10 µM; such cytotoxicity was lower than that exerted by equal concentrations of [Pt(*O*,*O*′-acac)(γ-acac)(DMS)] (Figure 5D).

## 4. Discussion

[Pt(*O*,*O*′-acac)(γ-acac)(DMS)], synthesized for the first time several years ago [7,8], has shown a high and rapid cytotoxic activity in endometrium, breast, neuroblastoma, and mesothelioma immortalized tumor cells [9,10,11,12,13]. Furthermore, [Pt(*O*,*O*′-acac)(γ-acac)(DMS)] is also able to consistently decrease the tumor mass of mouse xenograft model of breast, [14] mesothelioma [12,13] and renal cancers [14]. [Pt(*O*,*O*′-acac)(γ-acac)(DMS)] is a Pt(II) complex, having two acetylacetonate (acac) ligands and dimethylsulfide (DMS) coordinated to the metal, with the biological activities already cited above. Differently from cisplatin, for which the activity appears to be both genomic and non-genomic, [Pt(*O*,*O*′-acac)(γ-acac)(DMS)] shows a small reactivity with nucleobases and a characteristic reactivity with sulfur ligands [7,8]. This can make [Pt(*O*,*O*′-acac)(γ-acac)(DMS)] capable of acting intracellularly with different modalities from those caused by cisplatin. In the present study we used the renal cancer cells, Caki-1, that are considered to be a cisplatin-resistant cell line; in these cells [Pt(*O*,*O*′-acac)(γ-acac)(DMS)] is able to induce a strong cytotoxic effects both in vitro and in vivo [14]. Since Caki-1 cells hardly activate the apoptotic process, whereas [Pt(*O*,*O*′-acac)(γ-acac)(DMS)] always triggered apoptosis in all the cells tested, it seemed appropriate to determine the cellular effects induced by [Pt(*O*,*O*′-acac)(γ-acac)(DMS)] and compared with those obtained with cisplatin. On the other hand, a recent report showed that [Pt(*O*,*O*′-acac)(γ-acac)(DMS)] was able to induce autophagy pathway in neuroblastoma cells [18]. 

Furthermore, renal neoplasms are clinically resistant to Pt coordination complexes, not least to the cisplatin itself. Indeed, many chemotherapeutic agents have been used in the treatment of renal cell carcinoma in the advanced stage, but only floxuridine, 5-fluorouracil, and vinblastine have individually obtained results, though scarce [25]. More recently, mTOR and vascular endothelial growth factor receptor (VEGFR) inhibitors have been approved for the treatment of RCC [26,27,28,29]. Our recent results on Caki-1 cells [14] were confirmed here, with [Pt(*O*,*O*′-acac)(γ-acac)(DMS)] inducing cytotoxicity faster and greater than that induced by cisplatin. The different and important observation in renal cells was that the high mortality rate associated with [Pt(*O*,*O*′-acac)(γ-acac)(DMS)] was not due to apoptotic processes (caspases were not activated, poly ADP ribose polymerase (PARP) was not degraded, nor were DNA degradation or formation of condensed chromatin observed). Instead, the Caki-1 cells incubated with [Pt(*O*,*O*′-acac)(γ-acac)(DMS)] underwent a remarkable autophagic process that is not seen with the use of cisplatin. This conclusion is based on evidence that several autophagic markers are activated in the presence of [Pt(*O*,*O*′-acac)(γ-acac)(DMS)]. Autophagy does not always produce the same cellular effect, especially when it is triggered by antitumor drugs. Indeed, sodium selenite, [30] arsenic trioxide [31] and bortezomib are able to induce cell death through autophagy, whilst other studies showed that autophagy is significantly associated with cell survival and therapy resistance [32,33].

In our case, the inhibition of the autophagic process obtained with 3-MA showed an decrease in cell death due to [Pt(*O*,*O*′-acac)(γ-acac)(DMS)]. This data suggests that autophagy triggered in Caki-1 cells is a process fostering cell death. The MAPK JNK1/2 is known to be involved in the regulation of autophagy of cancer cells in response to pharmacological stress [34,35]. We show here that JNK1/2 was phosphorylated in [Pt(*O*,*O*′-acac)(γ-acac)(DMS)]-treated cells and that its inhibition blocked the [Pt(*O*,*O*′-acac)(γ-acac)(DMS)]-induced Beclin-1 increase. Beclin-1, a key component of the autophagosome nucleation complex, can interact with Bcl-2 to form Beclin-1/Bcl-2 complex, which functions as an inhibitor of autophagy [36].

The phosphorylation of Bcl-2 by JNK promotes Bcl-2 degradation and dissociation from Beclin-1, leading to induction of autophagy [37,38]. Consistently, JNK activation is also essential for autophagic cell death induced by anticancer agents [39,40]. We also made clear in this study that the PI3K/AKT/mTOR/p70S6K pathway is part of the transduction mechanism used by [Pt(*O*,*O*′-acac)(γ-acac)(DMS)] in inducing Caki-1 cell death. Several studies demonstrated that autophagy was often triggered by the inhibition of the PI3K/AKT/mTOR/p70S6K pathway concomitantly with the activation of the JNK pathway [41,42]. The PI3K/AKT/mTOR/p70S6K pathway has an important role in regulating cell survival and death, proliferation, and apoptosis.

In addition, various cellular signaling pathways, including AMPK and PI3K/AKT/mTOR/p70S6K, play a vital role in the process of autophagy [43,44,45]. The PI3K/AKT pathway acts as a positive regulator of the mTOR pathway, which serves as a negative regulator of autophagy in cancer cells [24], so that disruption of the PI3K/AKT/mTOR/p70S6K pathway by anticancer agents induces autophagy. This is used in in advanced RCC patients with multiple adverse risk features [46] where the inhibition of mTOR (notably by using rapamycin analogues) displays an overall progression-free survival advantage. More precisely, p-mTOR in abundant nutrient conditions phosphorylates and inactivates the Unc-51 like autophagy activating kinases (ULK1/2) protein complex, while the deprivation of nutrients brings about the dephosphorylation and activation of ULK1 and ULK2 and their location near the phagophore. Finally, anticancer drugs that inhibited the PI3K/AKT/mTOR axis putatively stimulated autophagy [47].

In conclusion, we have shown a drastic difference in Caki-1 cell response to cisplatin and [Pt(*O*,*O*′-acac)(γ-acac)(DMS)]. Cisplatin cytotoxicity was scarce, probably because Caki-1 cells are intrinsically able to resist and inhibit apoptosis [17,48]. The cytotoxic effect of [Pt(*O*,*O*′-acac)(γ-acac)(DMS)] was strong compared to that observed in other cancer cell lines, due to autophagy through a mechanism mediated by JNK and PI3K/AKT/mTOR/p70S6K pathways. [Pt(*O*,*O*′-acac)(γ-acac)(DMS)] may therefore represent an alternative approach to reducing renal cancer mass. However, to assess the general applicability of these findings on renal cell cancer patients, further experiments performed on more renal cancer cell lines are required.
